# Pigmentation Levels Affect Melanoma Responses to *Coriolus versicolor* Extract and Play a Crucial Role in Melanoma-Mononuclear Cell Crosstalk

**DOI:** 10.3390/ijms22115735

**Published:** 2021-05-27

**Authors:** Małgorzata Pawlikowska, Tomasz Jędrzejewski, Andrzej T. Slominski, Anna A. Brożyna, Sylwia Wrotek

**Affiliations:** 1 Department of Immunology, Faculty of Biology and Veterinary Sciences, Nicolaus Copernicus University, 87-100 Toruń, Poland; tomaszj@umk.pl (T.J.); wrotek@umk.pl (S.W.); 2 Department of Dermatology, Comprehensive Cancer Center, Cancer Chemoprevention Program, University of Alabama at Birmingham, Birmingham, AL 35294, USA; aslominski@uabmc.edu; 3 Laboratory Service of the VA Medical Center, Birmingham, AL 35294, USA; 4 Department of Human Biology, Faculty of Biology and Veterinary Sciences, Nicolaus Copernicus University, 87-100 Toruń, Poland; anna.brozyna@umk.pl

**Keywords:** melanoma, melanogenesis, pigmentation, *Coriolus versicolor* extract, co-culture, d-penicillamine, kojic acid, therapy resistance, necroptosis

## Abstract

Melanoma, the malignancy originating from pigment-producing melanocytes, is the most aggressive form of skin cancer and has a poor prognosis once the disease starts to metastasize. The process of melanin synthesis generates an immunosuppressive and mutagenic environment, and can increase melanoma cell resistance to different treatment modalities, including chemo-, radio- or photodynamic therapy. Recently, we have shown that the presence of melanin pigment inhibits the melanoma cell response to bioactive components of *Coriolus versicolor* (CV) Chinese fungus. Herein, using the same human melanoma cell line in which the level of pigmentation can be controlled by the L-tyrosine concentration in culture medium, we tested the effect of suppression of melanogenesis on the melanoma cell response to CV extract and investigated the cell death pathway induced by fungus extract in sensitized melanoma cells. Our data showed that susceptibility to CV-induced melanoma cell death is significantly increased after cell depigmentation. To the best of our knowledge, we are the first to demonstrate that CV extract can induce RIPK1/RIPK3/MLKL-mediated necroptosis in depigmented melanoma cells. Moreover, using the co-culture system, we showed that inhibition of the tyrosinase activity in melanoma cells modulates cytokine expression in co-cultured mononuclear cells, indicating that depigmentation of melanoma cells may activate immune cells and thereby influence a host anticancer response.

## 1. Introduction

Melanoma, the most aggressive skin cancer, arises through genetic mutations in melanocytes, the pigment producing cells that originate from the neural crest [1,2,3,4,5]. Although melanoma accounts for about 1% of all malignant skin tumors, it is the most rapidly increasing malignancy in the Caucasian population [2,6]. The high mortality rate among melanoma patients is related to its resistance to majority of therapeutic approaches during advanced stages (III and IV) of disease, to the location of the tumor (mucosal melanomas are traditionally more aggressive) and once the metastatic process occurred [2,7,8,9]. As opposed to the exponentially growing knowledge of melanoma biology, durable curative therapies are dramatically lagging behind in the treatment of metastatic melanoma. It seems that the interaction between the tumor microenvironment and epigenetic mutations enables phenotype switching, which, being a unique adaptive capacity of melanoma cells, may be responsible for overcoming the environmental stressors, among others the current therapies [10]. The observed resistance of melanoma cells is also linked to the presence of melanin pigment [5,7,11,12,13,14].

Melanogenesis is initiated by enzymatic hydroxylation of l-tyrosine to l-DOPA (l-dihydroxyphenylalanine), with further oxidation to DOPAquinone. Both reactions are catalyzed by tyrosinase [15]. The subsequent series of oxidation-reduction reactions leads to the formation of the polymorphous and multifunctional biopolymer, melanin [16,17,18].

Melanin possesses an antioxidative activity and acts as a free radical scavenger. Therefore, the major role of this pigment in normal melanocytes is the attenuation of ultraviolet radiation (UVR) penetration deep into the skin as well as protection against UV-induced DNA damage [16,17,18,19,20,21]. Nevertheless, melanin synthesis can also induce genotoxic and mutagenic effects via production of reactive intermediates during melanogenesis that could contribute to melanoma induction and progression [12,22,23,24]. Moreover, melanin pigment can impair the cytotoxic actions of anticancer agents and attenuate responsiveness of tumor cells to radiotherapy [7,11,13,14,25]. In addition, melanogenesis intermediates show potent immunosuppressive effects [13,24,26]. Melanogenesis also affects energy yielding metabolism and activates hypoxia-inducible factor 1-alpha (HIF-1α) [27,28,29,30]. Therefore, the melanin synthesis system acts as a double-edge sword, which, while protecting normal melanocytes from UVR and oxidative stress, can also increase melanoma cell resistance to different therapeutic modalities, including chemo-, radio- or photodynamic therapy [7,11,13,14,24,31,32].

The above-mentioned hypotheses have been confirmed in a number of experimental models of melanoma [14,30,33,34,35]. Recently, we have also shown that the presence of melanin pigment affects the melanoma cell response to bioactive components derived from the Chinese mushroom *Coriolus versicolor* (CV), which have potential immunomodulating and antineoplastic activities [36,37]. The main bioactive components of *C. versicolor* are the protein-bound polysaccharides (PBPs) derived from two different strains of CV: COV-1 (PSP) most commonly used in China and CM101 (polysaccharide krestin, PSK) used in Japan [37,38]. The analysis of therapeutic potential led to the adoption of products using CV extracts as an adjunct therapy for cancer patients already receiving chemotherapy or radiotherapy in China and Japan [37]; however, at the molecular level, the mechanisms of CV activity are not fully understood. Reports focusing on the anticancer and immunomodulatory effects of CV indicate the need to conduct rigorous studies to identify underlying mechanisms of action, especially for melanoma, where research data are limited. Previously, we showed that components of CV extract induce reactive oxygen species (ROS)- and c-Jun *N*-terminal kinase (JNK)-dependent cells death in non-pigmented melanoma cells. We revealed, however, that pigmented cells, in contrast to unpigmented counterparts, were not susceptible to the killing action of CV-derived components [36].

Because melanogenesis has the potential to affect the tumor behavior and thus melanoma therapy outcome, in this study, we have investigated the effects of suppression of melanin synthesis on the effectiveness of the killing of melanoma cells by CV extract. We evaluated the effect of the inhibitors of tyrosinase activity on melanoma cells’ sensitization to CV. Moreover, due to limited literature available regarding the influence of melanogenesis inhibition on the activity of immune cells within the tumor microenvironment, we investigated whether inhibition of the melanogenesis pathway may increase the co-cultured mononuclear cells reactivity against melanoma cells. Since the immune response is a very important factor in the antitumor defense [39], understanding the role of a tumor microenvironment in the prevention of tumor progression may bring new insights into melanoma targeting therapies.

This study is the first report providing evidence that inhibition of the melanogenesis process in the pigmented melanoma cells induces the RIPK1/RIPK3/MLKL-dependent necroptosis, a form of caspase-independent cell death, which represents one of the most promising anticancer therapeutic modalities to eliminate apoptosis-resistant tumor cells. Furthermore, we show that inhibition of tyrosinase activity in pigmented melanoma cells affects melanoma-mononuclear cell crosstalk and induces significant amplification of proinflammatory cytokines expression in co-cultured human peripheral blood mononuclear cells (PBMCs).

## 2. Results

### 2.1. Pigmented Melanoma Cells Are Resistant to Cytotoxic Activity of CV Extract

**Figure 1:**
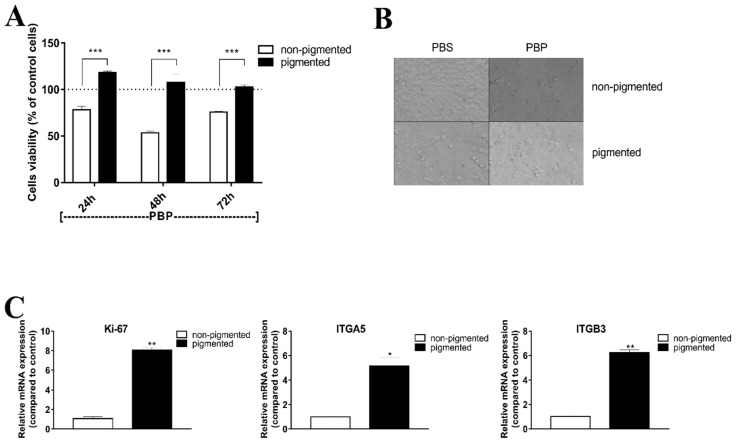
Pigmented melanoma cells are resistant to *Coriolus versicolor* (CV)-induced cytotoxic effect and express higher mRNA levels of proliferation marker and integrins compared to amelanotic cells. (**A**) The viability of pigmented and non-pigmented melanoma cells treated with CV extract at a concentration of 200 µg/mL. Data are reported as means ± SEM of three independent experiments, with six wells in each experiment. Asterisks indicate significant differences between non-pigmented and pigmented cells (*** *p* < 0.001). (**B**) Non-pigmented and pigmented melanoma cells treated with CV extract or PBS (control) for 72 h; 2D images were acquired using Olympus inverted microscope and analyzed using Olympus cellSens Dimension software (20 µm scale, 40× magnification). (**C**) mRNA expression levels of Ki-67, ITGA5, and ITGB3 in non-pigmented and pigmented melanoma cells determined by quantitative real-time PCR. Asterisks indicate significant differences between non-pigmented and pigmented cells (* *p* < 0.05, ** *p* < 0.01). Abbreviations: PBS—phosphate-buffered saline; CV—*Coriolus versicolor* extract.

Previously, we showed that pigmented and non-pigmented SKMel-188 melanoma cells differ in their sensitivity to bioactive compounds of CV during short-term exposure, up to 24 h [36]. Herein, using long-term exposure, we confirmed that pigmented melanoma cells are resistant to cytotoxicity induced by a CV extract. As shown in Figure 1A,B, the viability of pigmented melanoma cells, in contrast to their non-pigmented counterparts, was not affected by a fungus extract at the tested concentration. Because of the observed differences in response to CV between pigmented and non-pigmented cells, we examined in these cells the expression of two markers: metastasis-associated factor and proliferation marker, both of which were introduced to the diagnostics of melanoma in recent years. These parameters, among others [40], allow for establishing a course of treatment and may be useful in assessment of the melanoma therapy outcome. We checked whether pigmented and non-pigmented melanoma cells differ from each other in proliferation and invasion capacities at a molecular level. We assessed the mRNA expression of proliferation marker, Ki67, and the integrin alpha V beta 3 subunits, the integrin alpha 5 (ITGA5), and integrin beta 3 (ITGB3). As shown in Figure 1C, the mRNA expression of proliferation marker Ki67 in pigmented melanoma cells was higher compared to non-pigmented cells (8.07 ± 0.24 fold increase). Similarly, the mRNA levels of ITGA5 and ITGB3 were also altered in melanotic cells in comparison with cells with low melanin content (5.13 ± 0.71 and 6.22 ± 0.24 fold increase, respectively).

### 2.2. Inhibition of Melanogenesis Enhances Melanoma Sensitivity to the Killing Action of CV Extract

**Figure 2:**
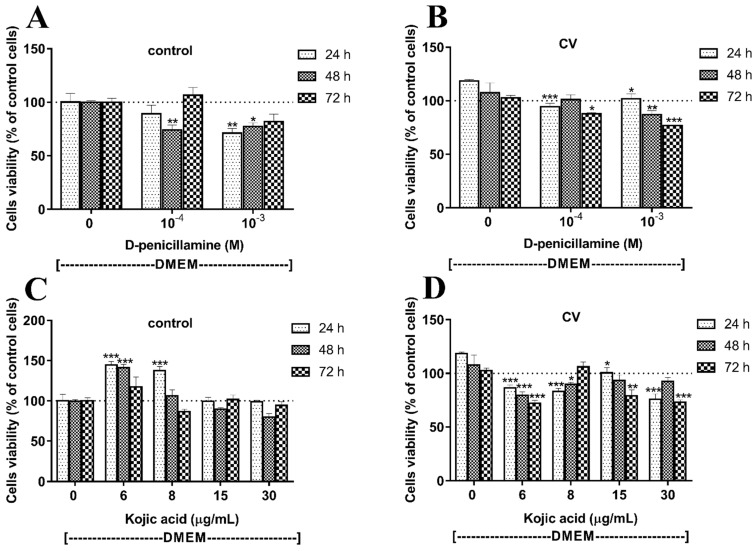
The inhibition of melanogenesis sensitizes SKMel-188 melanoma cells to the activity of *Coriolus versicolor* (CV) extract. (**A**,**C**) the viability of melanoma cells incubated with melanogenesis inhibitors: d-penicilammine (**A**) or kojic acid (**C**) for the indicated time periods. The “0” bars represent the control, non-stimulated and not incubated with d-pen or KA cells. (**B**,**D**) the effect of CV extract on melanoma cell viability previously exposed to melanogenesis inhibitors: d-pen (**B**) or KA (**D**). The “0” bars represent cells stimulated with CV extract, not incubated with d-pen or KA, respectively. Data are reported as means ± SEM of three independent experiments, with six wells in each experiment. Asterisk indicates significant differences between untreated samples (“0”) of a given time point with the treated cells of the same time point (* *p* < 0.05, ** *p* < 0.01, *** *p* < 0.001). Abbreviations: PBS—phosphate-buffered saline; CV—*Coriolus versicolor* extract; d-pen—d-penicillamine 10^−3^ M or 10^−4^ M; KA—Kojic acid 6, 8, 15 and 30 µg/mL.

**Figure 3:**
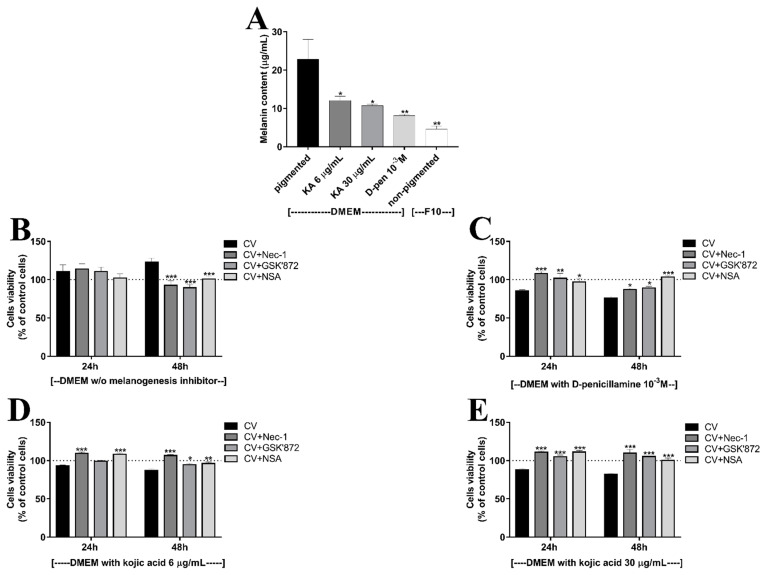
The inhibition of RIPK1, RIPK3, and MLKL abolishes the *Coriolus versicolor* (CV)-mediated cytotoxicity directed towards melanoma cells with inhibited melanogenesis; (**A**) the melanin content in SKMel-188 melanoma cells treated with d-pen or KA at indicated concentrations. Asterisks indicate significant differences between pigmented cells and depigmented/non-pigmented ones (* *p* < 0.05, ** *p* < 0.01); (**B**) the viability of pigmented melanoma cells treated with CV extract at a concentration of 200 μg/mL or co-treated with CV and Nec-1 (30 μM), GSK’872 (10 μM) or NSA (1.5 μM) for 24 and 48 h; (**C**–**E**) the viability of pigmented melanoma cells exposed to 10^−3^ M d-pen (**C**), 6 µg/mL KA (**D**) or 30 µg/mL KA (**E**) treated with CV at a concentration of 200 μg/mL or co-treated with CV and Nec-1 (30 μM), GSK’872 (10 μM) or NSA (1.5 μM) for 24 and 48 h. Data are reported as means ± SEM of three independent experiments, with six wells in each experiment. Asterisks indicate significant differences between CV-treated cells and cells co-treated with CV and necroptosis kinases inhibitors (* *p* < 0.05, ** *p* < 0.01, *** *p* < 0.001). Abbreviations: PBS—phosphate-buffered saline; CV—*Coriolus versicolor* extract; d-pen—d-penicillamine 10^−3^ M; KA—Kojic acid 6 or 30 µg/mL; RIPK1 - receptor interacting protein kinase 1; RIPK3 - receptor interacting protein kinase 3; MLKL - mixed lineage kinase domain-like protein; Nec-1—necrostatin-1; NSA—necrosulfonamide.

We next assessed whether inhibition of melanogenesis sensitizes melanoma cells to CV-induced cytotoxicity. For the selection of the optimal doses of tyrosinase inhibitors, the pigmented SKMel-188 cells were treated with various concentrations of d-penicillamine (d-pen) or kojic acid (KA) for 24–72 h and an MTT assay was performed. The results revealed that d-pen itself affected the SKMel-188 melanoma cells growth slightly, and the observed growth inhibition was dose-dependent; however, it was inversely related to time exposure to d-pen, especially at the 10^−3^ M concentration (Figure 2A). Tukey’s multiple comparison test showed no significant differences in cell viability after 72 h of incubation with d-pen at either concentration (10^−3^ M and 10^−4^ M) compared to control cells. KA had no inhibitory effects on melanoma cell viability; the concentrations of 6 and 8 µg/mL of KA were even upregulating cell proliferation after 24 and 48 h (Figure 2C). Next, we examined whether inhibition of melanogenesis by d-pen and KA sensitizes human melanoma cells to CV components (Figure 2B,D). The depigmented cells were exposed to fungus extracts for 24–72 h, and the cell viability was determined. As shown in Figure 2B, depigmentation using d-pen at a concentration of 10^−3^ M sensitizes cells to the cytotoxic action of CV. The observed effect was time-dependent. Similar effects were seen after treatment with KA. Melanoma cells pretreated with KA, especially at concentrations of 6 and 30 µg/mL, were sensitized to the cytotoxic activity of CV (Figure 2D). Therefore, further studies were performed with d-pen at a concentration of 10^−3^ M or KA at the concentrations of 6 and 30 µg/mL. These inhibitor concentrations strongly affected the melanization level of SKMel-188 cells (Figure 3A).

### 2.3. CV Extract Induces a Necroptotic Death Pathway in the Depigmented Cells

Next, we examined the potential death pathway induced in sensitized cells. Our previous data showed that CV bioactive components induce ROS- and JNK-dependent death of non-pigmented melanoma cells [36]. The observed cell death was independent of caspase and Bcl2 expression, and was mediated through induction of necroptosis, a form of programmed cell death [41]. To verify whether this pathway is also triggered by CV compounds in depigmented melanoma cells, we examined the potential involvement in this process of three kinases, including receptor interacting protein kinase 1 (RIPK1), RIPK3, and mixed lineage kinase domain-like protein (MLKL), which are the main executioners of necroptotic cell death. We co-cultured CV-stimulated depigmented melanoma cells with selected kinases inhibitors: necrostatin-1 (Nec-1), GSK’872 and necrosufonamide (NSA), which specifically target RIPK1, RIPK3, and MLKL activity, respectively [42,43,44]. The cytotoxicity assay confirmed that pigmented melanoma cells are resistant to CV and that necroptotic cell death is not induced (Figure 3B). Interestingly, the pretreatment of depigmented melanoma cells with Nec-1, GSK’872, or NSA abolished CV-mediated cell death (Figure 3C–E). The viability of melanoma cells depigmented with 10^−3^ M d-pen (Figure 3C), 6 µg/mL KA (Figure 3D), or 30 µg/mL KA (Figure 3E) increased after CV stimulation when the RIPK1, RIPK3, and MLKL kinases were inhibited. This observation confirms that CV extract induces necroptosis in depigmented melanoma cells.

### 2.4. Depigmentation of Melanoma Cells Partially Restores CV-Induced Intracellular ROS Generation

**Figure 4:**
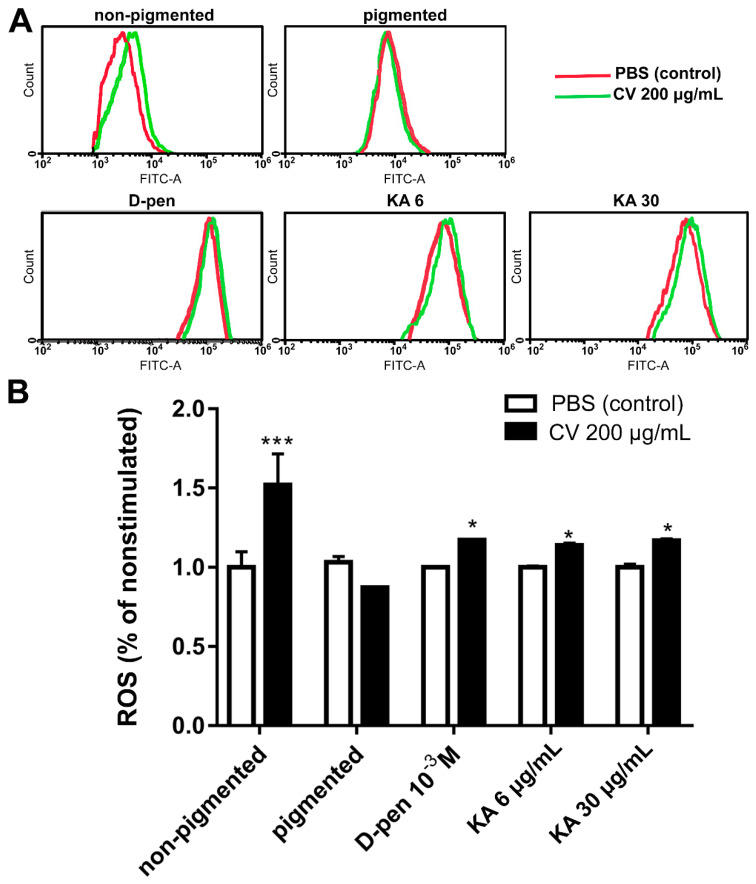
*Coriolus versicolor* (CV) extract induces reactive oxygen species (ROS) production in depigmented melanoma cells. Flow cytometry analysis visualized the amount of ROS generated in non-pigmented SkMel-188 cells (control for the study), pigmented, and melanogenesis inhibitors pre-treated SKMel-188, after CV (200 µg/mL) cells stimulation. (**A**) representative images of ROS generation shift upon CV stimulation of cells. The red line represents ROS production in cells incubated with PBS, green line refers to CV-stimulated group. (**B**) The relative levels of ROS in SKMel-188 cells are presented as fold change compared to PBS incubated cells. Data are reported as means ± SEM of three independent experiments. Asterisks indicate significant differences between control, PBS-treated cells, and CV-stimulated cells (* *p* < 0.05, *** *p* < 0.001). Abbreviations: non-pigmented—amelanotic SkMel-188 cells cultured in Ham’s F10 medium, pigmented—melanotic SkMel-188 cells cultured in DMEM medium, PBS—phosphate-buffered saline; CV—*Coriolus versicolor* extract; d-pen—d-penicillamine 10^−3^ M; KA—Kojic acid 6 or 30 µg/mL.

Since we have shown that the accumulation of intracellular ROS is involved in the induction of necroptosis in non-pigmented melanoma cells [36,41], we examined the ROS generation in depigmented melanoma cells following CV stimulation. The cells were treated with CV extract (200 µg/mL) or incubated with PBS (as a control) for 24 h, and then stained with 2′,7′ dichlorofluorescin diacetate (DCFH-DA) to measure total intracellular ROS (Figure 4A,B). The CV-induced ROS generation was then analyzed by flow cytometry. The treatment with CV in melanoma cells previously treated with the melanogenesis inhibitors, D-pen and KA, restored the production of ROS, which was totally inhibited in melanoma cells with the pigmented phenotype. Our results confirmed that melanin pigment inhibits ROS-generation in CV-stimulated melanoma cells, whereas non-pigmented cells, which are sensitive to CV extract, produce ROS (Figure 4A,B). The DFDH-DA analysis in depigmented cells showed that intracellular generation of ROS induced by CV compounds is partially restored when melanin synthesis is inhibited.

### 2.5. Pharmacological Inhibition of Melanogenesis in Melanoma Cells Increases the Reactivity of Co-Cultured Human PBMCs

**Figure 5:**
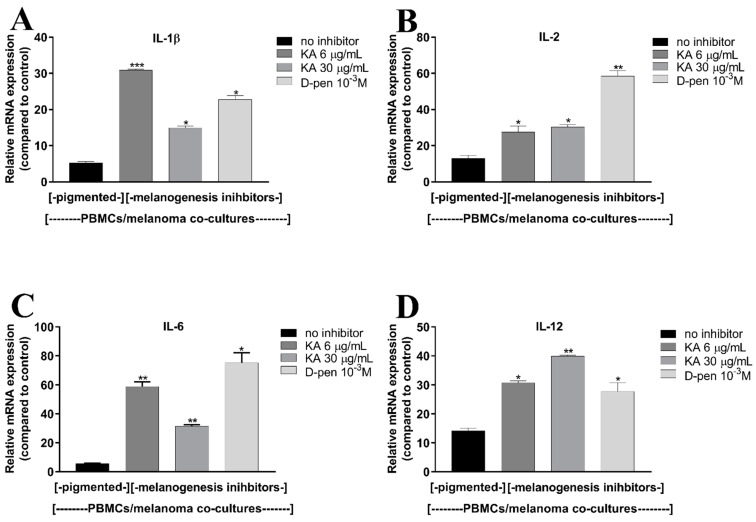
Inhibition of melanogenesis by d-pen or KA in melanoma cells affects the cytokine expression in co-cultured PBMCs. Pigmented melanoma cells were exposed to d-pen (10^−3^ M) or KA (6 or 30 µg/mL) for 72 h, then seeded on inserts with 0.4 µm pores. PBMCs were seeded in 24-well plates and activated with LPS (100 ng/mL); (**A**–**D**) the effect of melanogenesis inhibition in melanoma cells on IL-1β (**A**), IL-2 (**B**), IL-6 (**C**), and IL-12 (**D**) mRNA expression in co-cultured PBMCs determined by RT-qPCR. Asterisks indicate significant differences in mRNA expression of cytokines between PBMCs co-cultured with pigmented cells and depigmented ones (* *p* < 0.05, ** *p* < 0.01, *** *p* < 0.001). Abbreviations: PBMCs—peripheral blood mononuclear cells; d-pen—d-penicillamine 10^−3^ M; KA—Kojic acid 6- or 30 µg/mL; IL—interleukin.

To explore the potential inhibitory properties of melanogenesis directed towards the immune cell response, we assessed the immunocompetence of PBMCs in a co-culture system with pigmented and depigmented melanoma cells. Herein, we evaluated and compared the cytokine mRNA expression in PBMCs co-cultured with high melanin content SKMel-188 cells and those with an inhibited melanogenesis pathway. SKMel-188 cells for co-culture with PBMCs were propagated in DMEM medium or medium with the tyrosinase inhibitors, D-pen and KA, in order to inhibit melanin synthesis. As shown in Figure 5, melanogenesis inhibition affects the mRNA expression of all examined cytokines in the co-cultured PBMCs. The upregulation of IL-1β (Figure 5A), IL-2 (Figure 5B), IL-6 (Figure 5C), and IL-12 (Figure 5D) mRNA expression was significant in PBMCs co-cultured with melanoma cells depigmented by both tyrosinase inhibitors, compared to pigmented cells. Altogether, these data suggest that melanogenesis inhibits the reactivity of immune cells, which may lead to the immunosuppression.

## 3. Discussion

An extract from the *Coriolus versicolor* fungus and its bioactive components have been classified as a biological response modifiers (BRMs) with potential therapeutic application [38,45]. The in vitro and in vivo studies have demonstrated the immunostimulatory and anticancer properties of CV extract [37,46,47,48]; however, the molecular events underlying CV-induced mechanisms in different immune and cancer cell types are poorly understood. Several studies revealed that immunoregulatory effects of this extract in terms of the production of cytokines are related to the Toll-like receptor 4 (TLR-4) and nuclear factor κB (NF-κB) signaling pathways [38,49,50]. Other reports focusing on anticancer abilities of CV extract revealed that bioactive compounds of this fungus extract may influence cancer cells proliferation via cell cycle arrest [51,52,53]. As a mode of cell death, the induction of apoptosis has been shown for many cell types which was associated with caspase-3 activation via the mitochondrial pathway [51,54,55]. Recently, using the SKMel-188 melanoma cell line, which represents a unique research model that allows for obtaining the results independent of individual cell line properties resulting from differences at the DNA level and thus enabling the conducting comparative studies on the biology of both forms of melanoma, we have demonstrated that CV fungus compounds cause Bcl-2- and caspase-independent cell death in non-pigmented melanoma cells [36]. Subsequent studies have revealed that CV induces the programmed form of necrosis [41]. This cytotoxic activity of CV extract was observed only in non-pigmented melanoma cells, while pigmented cells were resistant to the anticancer activity of this agent. Herein, using the same human SKMel-188 melanoma cell line, in which the level of pigmentation can be controlled by l-tyrosine concentration in culture medium, we confirmed the previous findings. The long-term exposure of pigmented melanoma cells to fungus extract had no effect on their viability, which suggested that they were resistant to the cytotoxic activity of CV. These findings are in agreement with other reports [11,13,30,35] showing that actions of intermediates of melanogenesis and its final product, melanin pigment, can affect the outcome of melanoma therapy. Our results provide evidence that pigmented melanoma cells are also resistant to fungus-derived agents, which are used in the clinic as an adjunct therapy.

For years, the role of melanin in melanoma, especially the effect of melanin pigmentation on the invasiveness of melanoma cells, has been under extensive scrutiny. Herein, we demonstrate that highly pigmented melanoma cells are characterized by upregulated mRNA expression of the proliferation marker Ki67 and integrin alpha V beta 3 compared to non-pigmented cells. These results are in line with the report of Pinner et al. [56] showing that melanoma cancer cells, which form metastatic growth at secondary sites, are becoming highly pigmented and possess proliferative phenotype. Moreover, since there are data that induction of integrin signaling can, under some circumstances, save tumor cells from death-inducing effect of cytotoxic drugs [57], we presume that elevated expression of proliferation and invasiveness markers in pigmented melanoma cells may explain the problem of low response rate to CV therapy. However, further molecular studies are needed to verify this hypothesis.

Since melanin may protect malignant melanocytes against different treatment modalities [1,11,13,14,26,31,32], the inhibition of melanogenesis has been proposed as a potential adjuvant therapy of melanotic melanomas [24]. Therefore, in this work, we examined whether melanogenesis inhibition with the use of D-pen and KA would sensitize pigmented SKMel-188 cells to the cytotoxic effects of CV extract. D-pen, an FDA-approved D-cysteine-derivative with antioxidant, disulfide-reducing, and metal chelating properties, is used therapeutically in Wilson’s disease, lead poisoning, cystinuria, and rheumatoid arthritis [58,59]. However, this substantially non-toxic aminothiol, being a known copper-complexing agent, may also serve as a tyrosinase inhibitor. KA utilizes a similar mechanism of chelating the copper at the active site of tyrosinase and therefore prevents the conversion of the DOPA to DOPAquinone and subsequently to melanin [35]. Our results showed that D-pen itself induces a slight reduction of cell viability, whereas KA has no cytotoxic effects. The low doses of KA induced even upregulation of cell proliferation. These findings are in line with previous reports claiming that KA is nontoxic at doses below 100 µg/mL [60,61]. Kojic acid is only known to inhibit melanogenesis by direct inhibition of tyrosinase; however, it does not affect the expression of microphthalmia-associated transcription factor (MITF), which is engaged in proliferation and survival of melanoma cells [62]. Therefore, the melanoma cells can still proliferate, but the tyrosinase is not functional due to inhibition of KA [63]. In this study, we demonstrate that CV-resistance of melanoma cells correlates with the melanogenesis pathway and its inhibition by D-pen or KA increases SKMel-188 susceptibility to the fungus-derived agent. We postulate that the observed SKMel-188 cells sensitization to CV induced by both of the tested tyrosinase inhibitors may be related to a mechanism involving metal ion catalyzed H_2_O_2_-mediated oxidative stress, as has been observed in radio-chemo-sensitization of cancer cells [59]. However, additional experiments are required to determine the role of D-pen and KA in melanoma cell sensitization to CV-derived agents. Nevertheless, since the tested cells had the same genotype, our results clearly show that melanin and the process of melanin synthesis is responsible for melanoma cell resistance to CV.

Related to the mechanism of action, our results show that CV extract induce RIPK1/RIPK3/MLKL-dependent necroptosis, in the depigmented melanoma cells, which serves as an alternative mode of programmed cell death to eradicate apoptosis-resistant cancer cells. The use of the pharmacological inhibitors of the kinases RIPK1 (Nec-1), RIPK3 (GSK’872), and MLKL (NSA), which antagonize the necroptotic pathway and therefore rescue cells from necroptosis [64], abrogated the CV-induced cell death. To the best of our knowledge, this is the first report showing that CV induces necroptotic death of depigmented melanoma cells. Moreover, we previously showed that the cytotoxic effects of the fungus extract towards human non-pigmented melanoma cells were associated with deregulation of the cellular redox status [36]. We showed that non-pigmented melanoma cell death induced by CV extract is dependent on ROS production, which is regulated by c-Jun *N*-terminal kinase (JNK) activity [36]. Then, we discovered that the inhibitors of RIPK1, RIPK3, and MLKL kinases diminished the intracellular ROS generation triggered by CV compounds in non-pigmented melanoma cells [41]. Knowing that ROS plays a role in regulating necroptosis in many cell types [65], we sought to determine whether a blockade of the melanogenesis pathway would also affect ROS generation in CV-stimulated melanoma cells. As revealed by flow cytometry analysis of a DCFH-DA assay, the depigmentation of melanoma cells partially affected the ROS production triggered by CV extract when compared to pigmented cells. These results suggest that ROS generation may be one of the components of the necroptotic death pathway induced in depigmented melanoma cells following CV treatment. Since melanin is a known scavenger of reactive oxygen species, these findings may in part explain why the pigmented melanoma cells are resistant to CV treatment.

The tumor microenvironment is a heterogeneous ecosystem composed of a variety of cancer and immune cells that engage in an intricate crosstalk, thus shaping antitumor immunity [66]. Melanoma-associated antigens may activate B cells, T cells, and NK cells and thereby influence immune responses [67,68,69,70]. However, there are data indicating that the process of melanin synthesis in pigmented melanoma cells may affect the behavior of not only the melanoma cells but also its surrounding environment [26,71]. Since our and others data, suggest that melanogenesis inhibition sensitizes melanoma cells towards different therapeutic modalities, we tested whether inhibition of the melanogenesis pathway may increase mononuclear cells reactivity against melanoma cells. Indeed, using a co-culture system, we found that both d-pen and KA tyrosinase inhibitors strongly affect the activity of co-cultured PBMCs. Mononuclear cells co-cultured with depigmented melanoma cells have significantly upregulated mRNA levels of IL-1β, which is known for its antiproliferative effect on melanoma cells [72], and IL-2, the cytokine which is used as an immunotherapeutic agent in disseminated malignant melanoma [73,74,75], compared to those cultured with pigmented melanoma cells. Moreover, upregulation of expression of IL-12, an interleukin that has been shown to elicit significant anti-tumor activity in mice and humans [76,77,78], and IL-6, which acts as a growth inhibitor in early-stage melanoma [79,80], has also been observed in PBMCs co-cultured with depigmented SKMel-188. These results are in agreement with previous findings that L-DOPA, a major product released by pigmented cells, significantly decreased the cytokine mRNA levels in LPS-stimulated blood lymphocytes [13] and inhibited concovalin A- and LPS-induced immune activity of murine splenocytes and PBMCs [26]. They are also consistent with the report of Mohagheghpour et al. [81], who established that synthetic melanin at nontoxic concentrations effectively suppressed the production of cytokines, including IL-1β and IL-6, by human peripheral blood monocytes. Since cytokine expression by PBMCs is upregulated when depigmented cells are present in the co-culture, it is likely that melanogenesis suppresses the production of cytokines in PBMCs by interfering with posttranscriptional events, in a manner similar to that described previously [81]. However, additional experiments are required to clearly elucidate this molecular cascade. Nevertheless, our findings are in agreement with previous reports indicating that active melanogenesis in melanoma cells inhibits the host immune response and promotes tumor progression. The data presented above clearly suggest that the pigmented phenotype of cells attenuates CV-induced toxicity and, conversely, that inhibition of melanogenesis by tyrosinase inhibitors sensitize melanoma cells to the anticancer action of this fungus extract. The results are consistent with the known properties of melanin being a ROS scavenger [12,24] and with our previous findings showing that CV-derived components induce ROS-mediated cell death only of non-pigmented melanoma cells [36,41]. Moreover, using the co-culture system of PBMCs with melanoma cells with different melanin content, we confirmed that melanogenesis has potent immunosuppressive properties.

In conclusion, these results together with our latest reports undoubtedly contribute a significant enhancement of knowledge about biological meaningful of melanogenesis and its impact on melanoma cells therapy. Melanoma tumors are notoriously refractory to standard chemotherapeutic drugs [20] and solely focusing on killing these cells with antimitotic agents often leads to therapy-resistant mechanisms [10]. Therefore, additional pharmacological interventions using a CV extract to induce necroptosis as an alternate mode of programmed cell death would be beneficial in melanoma treatment.

Moreover, the resistance of pigmented melanoma cells in comparison to their non-pigmented counterparts to various therapies, including CV extract treatment, suggests that the presence of melanin synthesis pathway plays a significant role in rendering these cells less susceptible to cell death. Therefore, combining the inhibition of melanogenesis with CV extract application could be explored in preclinical studies as a valid therapeutic target for the adjunct management of pigmented melanomas.

## 4. Materials and Methods

### 4.1. Chemicals/Reagents

The CV extract was prepared from commercially available CV capsules (MycoMedica Company, Police nad Metují, Czech Republic) following previously described protocols [38,40,41]. The protein and carbohydrate contents in this extract were measured using phenol sulfuric acid and bicinchoninic acid assays, respectively. d-pen, KA (5-hydroxy-2-hydroxymethyl-1,4-pyrone), melanin, Nec-1, 3-(4,5-dimethyl-2-thiazolyl)-2,5-diphenyl-2H-tetrazolium bromide (MTT), 2′,7′-dichlorofluorescin diacetate (DCFH-DA), and lipopolysaccharide (LPS) were obtained from Sigma-Aldrich (Darmstadt, Germany). GSK’872 was purchased from R&D Systems (Minneapolis, MN, USA), and necrosulfonamide (NSA) was purchased from Tocris Bioscience (Bristol, UK). An EXTRACTME Total RNA Plus kit was obtained from Blirt company (Gdansk, Poland), and iScript Reverse Transcription Supermix for RT-qPCR, SSoAdvanced Universal SYBR^®^ Green Supermix, the PrimePCR™ SYBR^®^ Green Assay, human IL-1β (qHsaCID0022272), IL-2 (qHsaCID0015409), IL-6 (qHsaCID0020314), IL-12 (qHsaCID0007212), MKI67 (qHsaCID0011882), ITGA5 (qHsaCED0045939), ITGB3 (qHsaCED0045078) and ACTB (qHsaCED0036269) were purchased from Bio-Rad (Hercules, CA, USA). Ficoll–Paque Plus was bought from Amersham Biosciences (Piscataway, NJ, USA). All reagents for cell culture were purchased from Sigma-Aldrich unless stated otherwise.

### 4.2. Cell Culture

The human SKMel-188 melanoma cell line was a gift of Ashok Chakraborty, Yale University (New Haven, CT, USA). The cells were cultured at 37 °C in 5% CO_2_ in either Ham’s F10 (containing 10 μM l-tyrosine) or Dulbecco’s Modified Eagle’s Medium (DMEM) medium (containing 400 μM l-tyrosine) supplemented with 5% fetal bovine serum (FBS) and 1% antibiotics (penicillin/streptomycin/amphotericin) to maintain the non-pigmented versus pigmented phenotype, respectively. The media were changed every second day following protocols described previously [13,36,82]. PBMCs were derived from human blood samples of healthy volunteers. The cell isolation was performed using the density gradient centrifugation method. Briefly, the collected blood was diluted 1:1 (*v*/*v*) with PBS (pH 7.4) and carefully layered onto the separation medium (Ficoll–Paque Plus) in a 15 mL centrifuge tube. After centrifugation at 400× *g* for 35 min at RT, the PBMC fraction was collected. The cells were resuspended in RPMI 1640 medium with 10% FBS and antibiotics, activated with lipopolysaccharide (LPS; 100 ng/mL) for 24 h and used for the subsequent co-culture experiments.

### 4.3. Melanogenesis Inhibition and Depigmentation

Pigmented melanoma cells were originally treated with various concentrations of d-pen (10^−3^ M or 10^−4^ M) or KA (ranging from 6 to 30 µg/mL) to determine the least cytotoxic and most effective concentration. d-pen was dissolved in H_2_O (0.1 M) and further dilutions of the inhibitor were made in DMEM medium. KA was prepared as 60, 120, or 240 µg/mL stocks in PBS and further diluted with DMEM. The pigmented melanoma cells were cultured in the presence of d-pen or KA for 24 to 72 h. The optimal concentrations of tyrosinase inhibitors were then used for CV-induced cytotoxicity analysis and co-culture experiments.

### 4.4. Measurement of Melanin Content

SKMel-188 cells were plated in 12-well culture plates in Ham’s F10 (non-pigmented) or DMEM (pigmented) medium in the presence or absence of d-pen (10^−3^ M) or KA (6 or 30 µg/mL) for 72 h. The cells were then washed with PBS and 15 × 10^4^ of cells were sonicated and lysed in 150 μL of 1 M NaOH at 80 °C for 2 h. A total of 100 μL of the lysate was added in a 96-well microplate, and the absorbance was measured at 405 nm using Synergy HT Multi-Mode Microplate Reader (BioTek; Winooski, VT, USA). The absorbance was compared with a standard curve of synthetic melanin, and the melanin content was expressed as µg/mL.

### 4.5. Cell Viability Assay

The effects of the CV extract, melanogenesis inhibitors, alone or in combination with CV, as well as co-treatment with CV and inhibitors of necroptosis-related kinases on SKMel-188 cells viability were determined using an MTT assay. Briefly, in order to assess non-pigmented and pigmented melanoma cell responses to the fungus extract (200 µg/mL), 5 × 10^3^ cells/well were seeded into 96-well plates in Ham’s F10 or DMEM medium and, after a 24 h preincubation, they were stimulated with CV for 24 to 72 h. To evaluate the effect of melanogenesis inhibition, the cells were seeded in DMEM medium and exposed to graded concentrations of d-pen or KA for 24 to 72 h. The effect of CV extract on depigmented melanoma SKMel-188 was analyzed after 24, 48, or 72 h. The depigmented cells were co-treated with CV (200 µg/mL) and Nec-1 (30 μM), GSK’872 (10 μM) and NSA (1.5 μM) for the indicated periods of time to elucidate a cell death mechanism triggered by CV components in melanoma cells with an inhibited melanogenesis pathway. After treatments, all cells were incubated with a 0.5 mg/mL MTT solution for 3 h at 37 °C in an incubator, and the resulting purple formazan crystals were dissolved in DMSO. The optical density at 570 nm (with a reference wavelength of 630 nm) was measured using a Synergy HT Multi-Mode microplate reader. The cytotoxicity was determined as follows: absorbance of treated cells/absorbance of control cells × 100%.

### 4.6. Ki67 and Integrin Expression in Melanoma Cells

We evaluated the mRNA expression level of Ki67 and integrin alpha V beta 3 subunits (ITGA5 and ITGB3) in cells cultured in Ham’s F10 or DMEM medium for 24 h. Total RNA was extracted using an EXTRACTME Total RNA Plus kit, and first-strand cDNA was synthesized with the use of iScript Reverse Transcription Supermix for RT-qPCR according to the manufacturer’s protocol. qPCR was performed using SSoAdvanced Universal SYBR^®^ Green Supermix. Target mRNA expression was normalized to the reference gene, β-actin (ACTB). The primers used to detect ACTB, Ki67, ITGA5, and ITGB3 by qPCR were purchased and validated by Bio-Rad. RT-qPCR and qPCR were performed according to the MIQE guidelines [83], with a CFX Connect Real-Time PCR detection system (Bio-Rad), and the data analysis was performed using CFX Maestro™ software for CFX Real-Time PCR instruments. The comparative average cycle threshold method, 2^–ΔΔCT^ was used to analyze the mRNA expression levels and the values represented an *n*-fold difference relative to the calibrator. All data are presented in terms of relative mRNA and expressed as means ± standard error of the means (SEM). Differences were considered highly significant at *p* < 0.01 and significant at *p* < 0.05.

### 4.7. Measurement of Intracellular ROS

The non-pigmented, pigmented and depigmented melanoma cells (1 × 10^6^/well) were cultured in 6-well plates in the presence or absence of CV extract at a concentration of 200 µg/mL for 24 h. Then, the cells were harvested, washed twice with PBS, and co-incubated with serum-free Ham’s F10 medium containing 10 μM DCFH-DA for 30 min at 37 °C in the dark, according to the manufacturer’s instructions. At the end, cells were washed twice with PBS. DCF fluorescence distribution was detected by flow cytometry using BriCyte E6 (Mindray, Shenzhen, China) at an excitation wavelength of 488 nm and an emission wavelength of 525 nm.

### 4.8. Co-Cultures of PBMCs and Melanoma Cells for the qPCR Assay

To determine the effect of pigmentation status of melanoma cells on the modulation of cytokine production by PBMCs, a co-culture system was implemented. Prior to the co-culture, SKMel-188 cells, which were grown in DMEM medium, were depigmented using d-pen (10^−3^ M) or KA (6 or 30 µg/mL). Then, 1 × 10^5^ cells were seeded on inserts (0.4 μM pores) of 24-well plates (Corning, Tewksbury, MA, USA) and, after 24 h, the inserts were transferred to 24-well plates containing 0.5 × 10^6^ PBMCs per well. The co-culture was maintained for 24 h. The supernatants were aspirated and the RNA was isolated from PBMCs and RT-qPCR was done according to the procedures described above. The primers used to detect mRNA expression of IL-1β, IL-2, IL-6, and IL-12 by qPCR were purchased from and validated by Bio-Rad. 

### 4.9. Statistical Analysis

All values are reported as means ± SEM and were analyzed using analysis of variance followed by Tukey’s multiple comparisons test with the level of significance set at *p* < 0.05. Statistical analyses were performed with GraphPad Prism 7.0 (La Jolla, CA, USA). Comparisons between groups in qPCR for difference were performed using the unpaired Student’s *t*-test. A *p* value <0.05 was considered significant.
